# Quantitative proteomics study of the neuroprotective effects of B12 on hydrogen peroxide-induced apoptosis in SH-SY5Y cells

**DOI:** 10.1038/srep22635

**Published:** 2016-03-08

**Authors:** Lijun Zhong, Juntuo Zhou, Xi Chen, Yaxin Lou, Dan Liu, Xiajuan Zou, Bin Yang, Yuxin Yin, Yan Pan

**Affiliations:** 1 Medical and Health Analytical Center, Peking University Health Science Center, Beijing 100191, China; 2 Department of Pathology, School of Basic Medical Sciences, Peking University Health Science Center, Beijing 100191, China; 3 Institute of Systems Biomedicine, Department of Pathology, School of Basic Medical Sciences, Peking University Health Science Center, Beijing, 100191, China; 4 Beijing Key Laboratory of Tumor Systems Biology, Beijing, 100191, China; 5 Department of Pharmacology, School of Basic Medical Sciences, Peking University Health Science Center, Beijing 100191, China

## Abstract

B12 belongs to the coumarin class of compounds that have been shown to have various physiological and pharmacological activities including anti-inflammatory, antibacterial, and antioxidant. In the present study, we characterised the neuroprotective effects of B12 against H_2_O_2_-induced neuronal cell damage in SH-SY5Y cells. Protein expression profiling in combination with pathway analysis was deployed to investigate the molecular events associated with the neuroprotective effects in human neuronal cells using a label-free quantitative proteomics approach. A total of 22 proteins were significantly differentially expressed in H_2_O_2_-damaged cells with or without B12 treatment. Bioinformatics analysis using the Cytoscape platform indicated that poly pyrimidine tract binding protein 1 (PTBP1) was highly associated with the protective effect, and western blotting verified that PTBP1 was up-regulated in H_2_O_2_ + B12 treatment group, compared with the H_2_O_2_ treated group. PTBP RNAi experiments knocked down PTBP expression, which cancelled out the protective effect of B12 on cell viability. Thus, we infer that B12 neuroprotective activity involves up-regulation of PTBP1 and its associated signalling networks following H_2_O_2_-induced apoptosis in SH-SY5Y cells. B12 or related compounds may prove to be useful therapeutic agents for the treatment of neurodegenerative diseases such as Alzheimer’s and Parkinson’s.

Neurodegenerative diseases such as Parkinson’s (PD) and Alzheimer’s (AD) are characterized by the progressive loss of specific neuronal cell populations, and are associated with the formation of protein aggregates^1^,^2^,^3^. A common feature of these diseases is a connection with oxidative stress, which leads to the dysfunction or death of neuronal cells and contributes to disease pathogenesis^3^. Oxidative damage is generated following cell lysis and a burst of oxidative activity that results in an accumulation of oxygen free radicals that attack proteins, nucleic acids, and lipid membranes, thereby disrupting cellular function and integrity^4^. Hydrogen peroxide (H_2_O_2_) is among the main reactive oxygen species (ROS) and is produced in redox processes^5^,^6^. Oxidative stress manifests its toxic effects through different pathways, and knowledge of these pathways could assist the development of a number of potential therapeutic strategies that target neurodegenerative diseases^4^.

Proteomics, the collective study of all expressed proteins in cells, tissues, or biological fluids at a given time or under given conditions, reveals information on not only the individual components (proteins), but also their interplay in complexes, signalling pathways, and network modules associated with specific biochemical functions^7^,^8^,^9^. Proteomics studies generally involve high-throughput experimental platforms such as liquid chromatography coupled with tandem mass spectrometry (LC–MS/MS)^10^,^11^,^12^ that measures the abundance of proteins or peptides in different biological conditions by assaying thousands of proteins from complex biological samples simultaneously^13^. Shotgun proteomics using isotopic labelling can achieve accurate quantitative measurements^14^, but label-free quantitative proteomics is growing in popularity because it is reliable, versatile, and is a cost-effective alternative to labelled quantitation^15^.

Various compounds have antioxidant activity, including vitamin C, ubiquinone lipoic acid, β-carotene, creatine, melatonin, and curcumin^1^. Sung-Soo Kim *et al*.^16^ found that 3,5-dicaffeoylquinic acid (3,5-diCQA) has neuroprotective properties against neuronal cell death caused by H_2_O_2_-induced oxidative stress that operate by suppressing caspase-3 activation and restoring glutathione (GSH) levels. Similarly, Venuprasad and colleagues^17^ showed that a hydroalcoholic extract of holy basil (*Ocimum sanctum)* ameliorates H_2_O_2_-induced neuronal damage by stimulating the antioxidant defence mechanism, and could potentially be used to treat oxidative stress-mediated neuronal disorders. B12 (*4-*methyl-*7-*hydroxy-8-(1-hydroxyethyl) coumarin; CN patent No. 201210042735.1) is a compound belonging to the coumarin class that have been shown to possess anti-inflammatory, antibacterial, and antioxidant activities. In the present study, we characterised the neuroprotective effects of B12 against H_2_O_2_-induced neuronal cell damage in SH-SY5Y cells. Moreover, we used protein expression profiling combined with pathway analysis to investigate the molecular events associated with the protective effects, and gained insight into the underlying mechanisms of oxidative damage and neuroprotection.

## Results

### Neuroprotective effects of B12 on H_2_O_2_-treated SH-SY5Y cells

**Figure 1:**
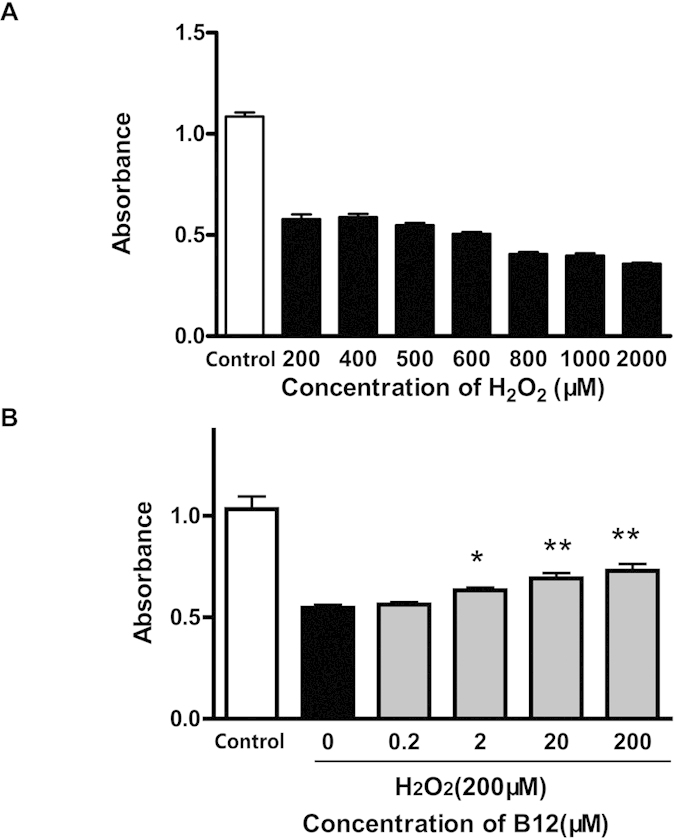
Cell proliferation following H_2_O_2_ or B12 treatment. (**A**) Cell proliferation of SH-SY5Y cells was monitored following H_2_O_2_ treatment at different doses. Death rate increases with increasing H_2_O_2_ concentration (*P < 0.05; **P < 0.01, compared with control group). (**B**) Neuroprotective effects of B12 are apparent at a concentration above 2 μM, and the cell survival rate is 75% at an H_2_O_2_ concentration of 200 μM (n = 6). Data are mean ± S.E.M. Experiments were repeated in triplicate.

Cell proliferation was observed in SH-SY5Y cells after treatment with H_2_O_2_ at a range of doses, and cell number decreased with increasing H_2_O_2_ concentration. A concentration of 200 μM H_2_O_2_ decreased cell number by 50%, and this was chosen for subsequent experiments on the neuroprotective effects (Fig. 1A). Clear differences in cell survival rate were apparent following treatment with B12, and survival varied with B12 concentration in a dose-dependent manner. The neuroprotective effects of B12 were prevalent at >2 μM, and a 75% cell survival rate was achieved at 200 μM H_2_O_2_ (Fig. 1B). Concentrations of 200 μM H_2_O_2_ and 20 μM B12 were subsequently used to analyse protein expression in label-free proteomics experiments.

### Protein expression analysis

**Figure 2:**
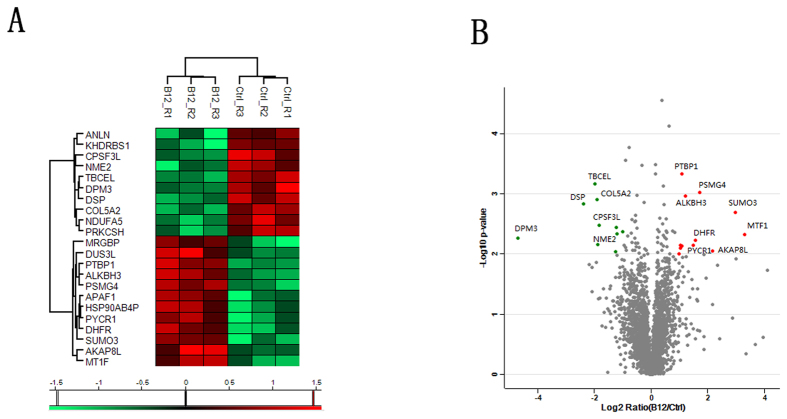
Protein expression profiling using a label-free quantitative proteomics approach. (**A**) Unsupervised hierarchical clustering analysis of 22 genes that are differentially expressed following treatment with H_2_O_2_ + B12 (B12 group; n = 3) compared to treatment with H_2_O_2_ alone (Ctrl group; n = 3). Gene names are listed on the left, while cell culture samples are indicated on top. The colour key beneath the heatmap indicates the level expression level (red = up-regulation, green = downregulation). (**B**) Scatter plots of fold change (x-axis) against log p-value (y-axis) of all quantified proteins. Up- and down-regulated proteins are coloured red and green, respectively.

A total of 4,969 proteins were identified using the shotgun method, and after applying the label-free algorithm (repeated measurement of at least two peptides of each protein in all six samples), 3,505 proteins were successfully quantified. A logarithmic transformation (base 2) of the LFQ intensity of proteins was pre-performed prior to the identification of differentially expressed proteins (Fig. S2). Statistical analysis with Perseus software was performed to select proteins that were differentially expressed following B12 treatment, using the following criteria: fold change >2, p-value <0.01 (using the Student’s *t*-test). As a result, we identified 22 significantly differentially expressed proteins in the H_2_O_2_ + B12 treatment group (P < 0.01; shown as B12 in figures) compared with the H_2_O_2_ treatment group (shown as Ctrl in figures). Of these, 12 proteins were up-regulated and 10 proteins were down-regulated (Table 1). Information on protein intensity and RSD ratio is shown in Table S1. Hierarchical clustering analysis of the 22 significantly differentially expressed proteins was performed (Fig. 2A), and scatter plots were constructed to determine the fold change in protein expression (x-axis = log2 Ratio (B12/Ctrl), y-axis = log p-value; Fig. 2B). Additional MS and Venn diagram data, as well as scatter plots, are shown in Figs S3, 4). The Venn diagram (Fig. S3) shows the number of identified proteins in the three replicates, including their overlap in the replicates. Scatter plots of protein intensity from all replicates against the Pearson correlation coefficient (Fig. S4) showed a higher correlation within the same group than between different groups.

### Bioinformatics analysis

**Figure 3:**
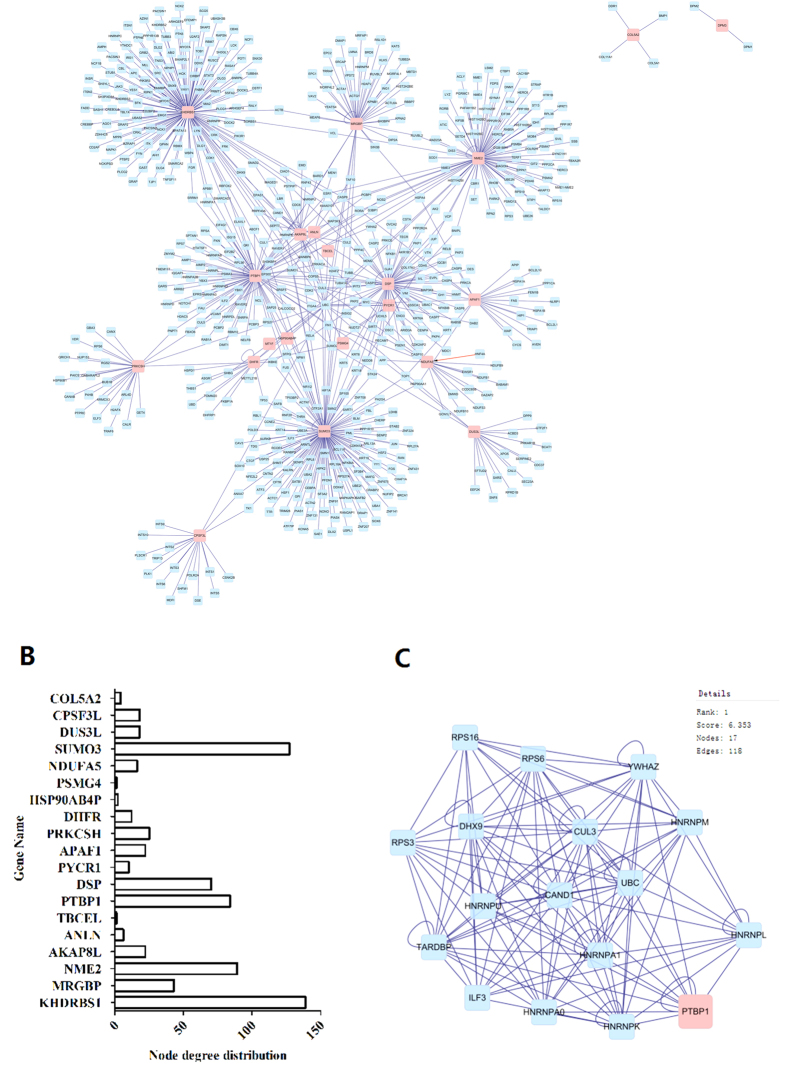
Network analysis using Cytoscape. (**A**) Integrated regulatory network constructed using Cytoscape. The network includes two types of nodes: differentially expressed proteins identified from protein profiling (pink squares), and their interaction targets (blue squares) derived from six publically available databases. (**B**) Node degree distribution of differentially expressed proteins. (**C**) Cluster analysis ranking of the top hits in the B12 regulatory network using the cluster search algorithm MCODE.

**Figure 4:**
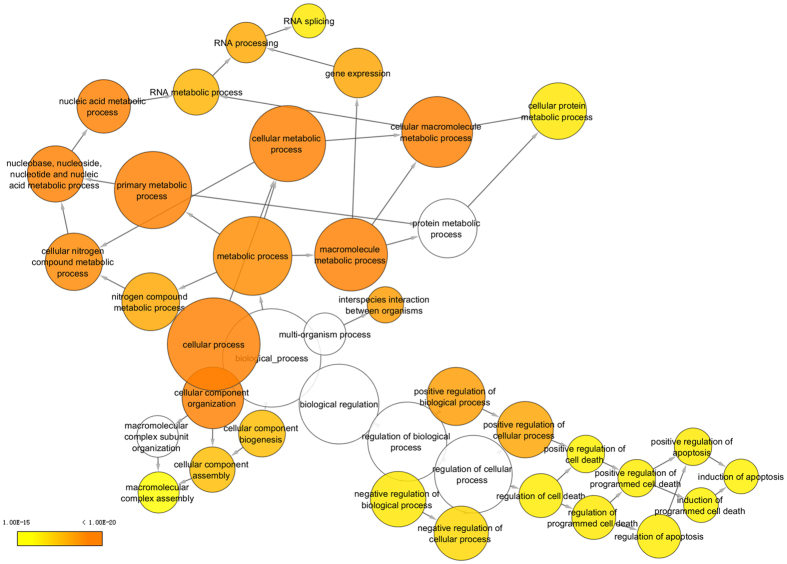
Gene Ontology (GO) terms associated with proteins interacting with the 22 differentially expressed proteins. The colour gradient of the cluster distribution network of the GO biological process associated with the neuroprotective effects of B12 shows the p-value of each cluster associated with the term, while the darker (orange) colour indicates a lower p-value.

To construct a protein-protein interaction network associated with B12 regulation, we matched the 22 significantly differently expressed proteins with regulatory data in six publically available databases (DIP, BIOGRID, HPRD, BIND, MINT and INTACT). The resulting network was visualized with Cytoscape 3.1.1 and included 614 nodes and 712 edges (Fig. 3A). The node degree distribution is shown in Fig. 3B. The cluster search algorithm, which considers highly interconnected dense regions within a network, was used to rank network proteins. Protein poly pyrimidine tract binding protein 1 (PTBP1), which was up-regulated in B12-treated cells, was the highest ranked (Fig. 3C), indicating a potential role in the neuroprotective effects of B12. Gene ontology analysis was then performed using the BinGO plugin in Cytoscape, and genes influenced by B12 were highly correlated with cellular macromolecule metabolic process, cellular metabolic process, nucleic acid metabolic process and gene expression biological process categories (Fig. 4; Table S2). PTBP1 was also associated with these biological processes (Table S2). The top 10 biological processes are listed in Table S2.

### Western blot verification of differentially expressed protein PTBP1

**Figure 5:**
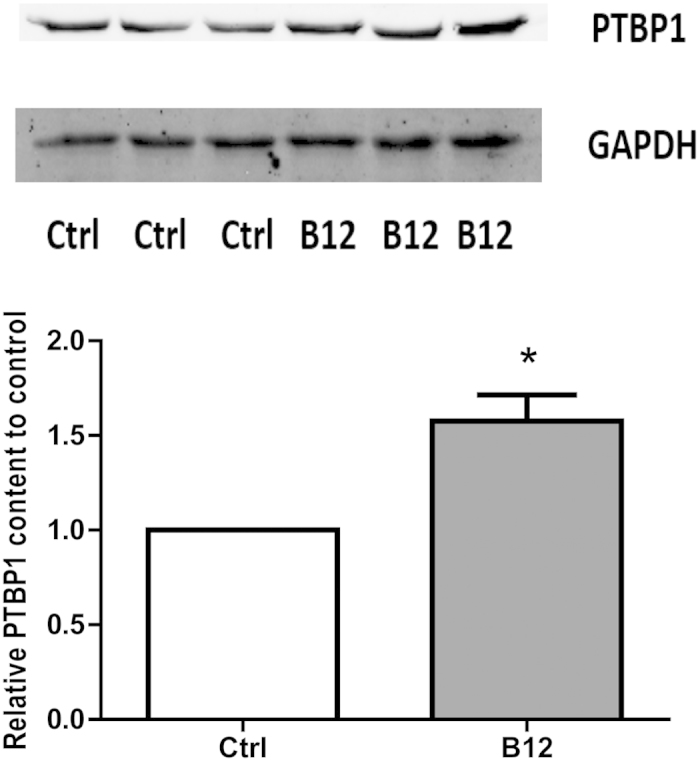
Western blotting of PTBP1 expression following treatment with H_2_O_2_ + B12 or H_2_O_2_ alone. The band intensity shows that treatment with H_2_O_2_ + B12 (B12) results in up-regulation of PTB1 compared with H_2_O_2_ alone (Ctrl) (*P < 0.05; n = 3; mean ± S.E.M). Experiments were repeated in triplicate.

In view of the important role of PTBP1 in the neuroprotective effect of B12, we used western blotting to verify expression in treatment and control groups, and found that PTBP1 was significantly up-regulated in the H_2_O_2_ + B12 treatment group (Fig. 5).

### PTBP1 knockdown confirmed its involvement in the neuroprotective effect of B12

**Figure 6:**
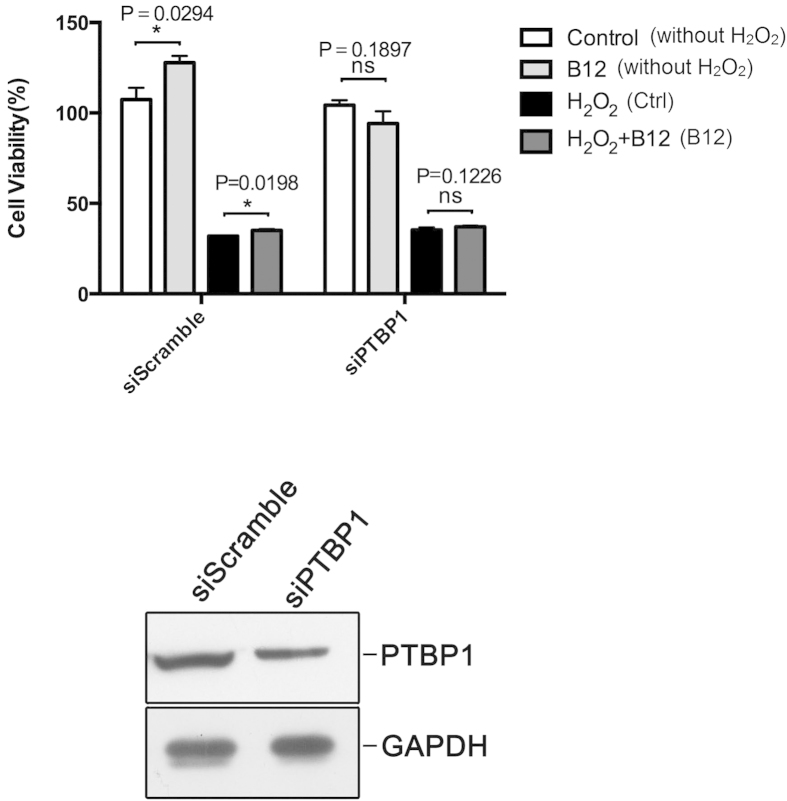
PTBP1 contributes to the neuroprotective effects of B12. (**A**) Viability of SH-SY5Y cells treated with or without H_2_O_2_ and B12 after PTBP1 knockdown. (**B**) Immunoblot of PTBP1 in SH-SY5Y cells after transfection with PTBP1siRNA to validate the knockdown level. (*P < 0.05; n = 3; mean ± S.E.M). Experiments were repeated in triplicate.

After validation of PTBP1 expression by western blotting, we further investigated its role using knockdown experiments. Following treatment with 200 μM H_2_O_2_ and 20 μM B12, clear differences were observed in the cell survival rate between the PTBP1 knockdown (siPTBP1) and control (siScramble) groups (Fig. 6A). Specifically, cell survival was increased in the siScramble group but not the siPTBP1 group, and the number of cells was also increased in cells treated with B12 without H_2_O_2_, whereas B12 had no effect on the PTBP1 knockdown group. Knockdown of PTBP1 was confirmed by immunoblotting of transfected cells (Fig. 6B).

## Discussion

H_2_O_2_ is a ubiquitous ROS that is produced in cells by superoxide dismutases as a metabolite of the superoxide anion (O_2_^2−^). H_2_O_2_ is a relatively stable and neutrally charged compound that can readily cross cell membranes, and these properties have prompted researchers to propose that H_2_O_2_ may be involved in oxidative stress signaling^3^. In the present study, we used H_2_O_2_ as a mediator of oxidative stress in the human neuroblastoma SH-SY5Y cell line, and H_2_O_2_-induced cell damage at a concentration of 200 μM or higher. We discovered that B12 could significantly protect cells from damage induced by H_2_O_2_-induced oxidative stress in a dose-dependent manner. B12 could therefore be used as a drug to protect cells from oxidative damage, and we further investigated the mechanism underlying the neuroprotective effects.

The results of quantitative proteomics and western blotting revealed that PTBP1 protein levels were up-regulated in cells in which B12 was protecting against oxidative stress-induced apoptosis. PTBP1 belongs to a subfamily of ubiquitously expressed heterogeneous nuclear ribonucleoproteins (hnRNPs), which are RNA-binding proteins that form a complex with heterogeneous nuclear RNA (hnRNA). These proteins are associated with pre-mRNAs in the nucleus and appear to influence pre-mRNA processing and other aspects of mRNA metabolism and transport. Cote *et al*.^18^ observed PTBP1 protein degradation in H_2_O_2_-treated breast cancer cell lines using western blotting. A recent study by King *et al*.^19^ showed that PTBP1 is involved in specific protein sub-complexes that control gene expression during apoptosis induced by TNF-related apoptosis-inducing ligand (TRAIL).

PTBP1 knockdown experiments provided more evidence of the important and potentially pivotal role of PTBP1 in the neuroprotective effects of B12, since B12 could no longer rescue cells damaged by oxidative stress after PTBP1 knockdown. Furthermore, the number of cells increased in cells treated with B12 in the absence of H_2_O_2_, indicating an involvement not only in protecting against oxidative stress, but in cells not damaged by H_2_O_2_, although this requires further study. In summary, we can infer that pre-mRNA processing is involved in the neuroprotective effects of B12, and expression of PTBP1, the main target of B12, is required to mediate resistance against H_2_O_2_-induced oxidative damage.

Comprehensive analysis of protein composition, post-translational modifications, and protein dynamics are important goals in cell biology. High performance liquid chromatography mass spectrometry (LC-MS) has established itself as a powerful tool for proteomics studies^20^. Although comprehensive gene expression profiles can be precisely and comprehensively quantified using transcriptomics approaches, transcript levels often show only a modest correlation with the abundance of their corresponding proteins^21^. Moreover, proteins are usually more directly responsible for the regulation of cellular pathways than mRNA transcripts. Thus, protein expression profiling is critical for elucidating the mechanisms underlying the cellular response to specific treatments or drugs^22^. Choi *et al*.^23^ used an LC-MS strategy to separate and identify components of the secretory proteome in adipocytes that are responsive to oxidative stress, and their results indicated that oxidative stress significantly down-regulated MMP-2 secretion and up-regulated TIMP-2 secretion. MS-based proteomic profiling experiments can therefore provide useful and important information to improve our understanding of complex biological processes.

Before analysing the proteomic data to identify significantly differentially expressed proteins in the present study, we first measured the expression of glyceraldehyde-3-phosphate dehydrogenase (GAPDH), a house keeping protein commonly used as an internal control in western blotting experiments. The ratio of GAPDH in B12-treated and control cells (B12/Ctrl) was 1.04, which confirmed the reliability of our label-free quantitative proteomics approach for expression profiling. Our results (Table 1) showed that 12 proteins (MT1F, SUMO3, AKAP8L, PSMG4, DHFR, PYCR1, ALKBH3, DUS3L, PTBP1, APAF1, HSP90AB4P, MRGBP) were up-regulated and 10 proteins (PRKCSH, KHDRBS1, NDUFA5, ANLN, CPSF3L, NME2, COL5A2, TBCEL, DSP, DPM3) were down-regulated in the H_2_O_2_ + B12 treatment group. Many of these proteins are reportedly involved in oxidative stress and apoptosis. SANG *et al*.^24^ reported oxidative stress-induced down-regulation of SUMO3 at the transcription level, while Yang *et al*.^25^ observed activation of SUMO2/3 conjugation as a protective response following brain ischemia/stroke. Our results, together with previous reports, suggest B12 induces the expression of SUMO3 at both mRNA and protein levels, and protects cells from H_2_O_2_-induced oxidative damage, further linking sumoylation to neuroprotection. Mejia *et al*.^26^ reported that Sam68 (KHDRBS1) is recruited into stress granules in response to oxidative stress. In summary, our results provide valuable information on proteins involved in oxidative stress and mediating against oxidative damage.

Interaction networks are useful for studying complex systems in multiple disciplines including economics, sociology, and biology^27^, and can assist the interpretation of the results of proteomics studies. Analysis of proteomics data at the pathway level has become increasingly popular, as demonstrated by Borroto-Escuela *et al*.^28^ who used network analysis to study molecular integration in G protein-coupled receptors (GPCRs) to elucidate the overall architecture of GPCR heteromers. Functional network analysis combined with gene ontology revealed that the vast majority of the significantly differentially expressed proteins identified in the present study were involved in cellular macromolecule metabolic process, cellular metabolic process, nucleic acid metabolic process, cellular component organization and gene expression categories (Fig. 4; Table S2). It was reported that both triethylene glycol dimethacrylate (TEGDMA) and 2-hydroxyethyl methacrylate (HEMA) were able to activate the coordinated network of the mitogen-activated protein kinase (MAPK) and phosphatidylinositol 3-kinase (PI-3K) pathways^29^. In another study, some of the activated proteins detected functioned in signal transduction pathways associated with the regulation of specific transcription factors^14^. In the present study, the key regulatory protein PTBP1 was associated with cellular macromolecule metabolic process, nucleic acid metabolic process and gene expression biological process categories, consistent with an important role in the neuroprotective effects of B12. Our network analysis and gene ontology results also highlighted other biological processes that are involved in oxidative stress and the anti-oxidative response. Our results provide information on the mechanism underlying the response of cells to H_2_O_2_-induced oxidative stress, and this information may prove useful for the development of treatments for neurodegenerative diseases, although further studies are required to determine the exact mechanism.

## Conclusions

The present study indicated that B12 (at concentrations higher than 2 μM) can protect cells from H_2_O_2_-induced cell damage by inducing specific cellular responses at the protein level. Of the differentially expressed proteins, the pre-mRNA processing protein PTBP1 was found to be pivotal. Functional network analysis and gene ontology indicated the neuroprotective effects of B12 are associated with the metabolism of macromolecules and other cellular metabolic processes. Our results provide information on the mechanisms underlying the cellular response to H_2_O_2_-induced oxidative stress, which may prove to be useful for treating chronic neurodegenerative diseases such as Parkinson’s and Alzheimer’s.

## Materials and Methods

### Materials

3-(4,5-dimethylthiazol-2-yl)-5-(3-carboxymethoxyphenyl)-2-(4-sulfophenyl)-2H-tetrazolium (MTS), ammonium bicarbonate, sodium deoxycholate, iodoacetamide, and dithiothreitol were purchased from Sigma (St. Louis, MO, USA). Tris-(2-carboxyethyl) phosphine was acquired from Thermo Scientific (Rockford, Il, USA). Modified sequencing-grade trypsin was obtained from Promega (Madison, WI, USA). All mobile phases and solutions were prepared with HPLC grade solvents (i.e. water, acetonitrile, methanol, and formic acid) from Sigma Aldrich. All other reagents were from commercial suppliers and of standard biochemical quality.

### Cell culture and treatment

Cell culture: Human neuroblastoma SH-SY5Y cells were maintained in minimum essential medium (MEM) supplemented with 10% fetal calf serum, 100 U/ml penicillin and 100 U/ml streptomycin in a humidified atmosphere with 5% CO_2_ at 37 °C.

H_2_O_2_ treatment: SH-SY5Y cells were placed in plates and pretreated with various concentrations (200, 400, 500, 600, 800, 1000 and 2000 μM) of H_2_O_2_ for 6 h. Control cells were grown in MEM containing 0.5% fetal calf serum, but without H_2_O_2_.

B12 treatment: SH-SY5Y cells were placed in plates and pretreated with various concentrations (0.2, 2, 20 and 200 μM) of B12 for 12 h, followed by exposure to 200 μM of H_2_O_2_ for another 24 h. To induce oxidative stress, H_2_O_2_ was freshly prepared from 30% stock solution prior to each experiment. Control cells were grown in MEM containing 0.5% fetal calf serum, but without H_2_O_2_ and B12.

### Analysis of cell viability

SH-SY5Y cells were treated, and cell viability was determined using the conventional MTS reduction assay. Briefly, after treatment, 40 μl of MTS (2 mg/ml in PBS) was added to each well, and cells were incubated at 37 °C for 4 h. Supernatants were aspirated carefully, and the absorbance at 490 nm was measured (in triplicate) with a microplate reader (BIO-RAD Model 3550, CA, USA). Experiments were repeated three times.

### Sample preparation for shotgun analysis

In this study, cells treated with 200 μM H_2_O_2_ and no B12 were chosen as the control group (H_2_O_2_ treated group). Cells treated with 200 μM H_2_O_2_ and 20 μM B12 comprised the H_2_O_2_ + B12 treatment group. After determination of the density of the harvested SH-SY5Y cells, aliquots containing 1000 to 10000 cells were collected and placed in individual tubes. Cell pellets were flash frozen in liquid nitrogen and stored at −80 °C. Proteins were extracted with RIPA lysis buffer, and each group was analyzed in triplicate. A workflow flowchart is shown in Fig. S1. Protein samples (200 μg) from each group were processed according to the manufacturer’s protocol for filter-aided sample preparation (FASP)^30^. Proteins were concentrated using Vivacon 500 filtration tubes (Cat No. VNO1HO2, Sartorius Stedim Biotech, UK), mixed with 100 μL of 8 M urea in 0.1 M Tris/HCL (pH 8.5), and centrifuged at 14000 g for 15 min at 20 °C. This step was performed twice, after which 10 μL of 0.05 M Tris-(2-carboxyethyl) phosphine (TCEP) in water was added to the filters, and samples were incubated at 37 °C for 1 h. Then, 10 μL of 0.1 M iodoacetamide (IAA) was added to the filters, after which the samples were incubated in darkness for 30 min. Filters were washed twice with 200 μL of 50 mM NH_4_HCO_3._ Finally, 4 μg of trypsin (Promega, Madison, WI) in 100 μL of 50 mM NH_4_HCO_3_ was added to each filter. The protein to enzyme ratio was 50:1. Samples were incubated overnight at 37 °C and released peptides were collected by centrifugation.

High pH reverse phase chromatography was performed using the Dionex Ultimate 3000 Micro Binary HPLC Pump system^31^. The digested peptide mixture was reconstituted with 600 μL buffer A (20 mM ammonium formate in water, pH 10) and loaded onto a 2.1 mm × 150 mm Waters BEH130 C-18 column containing 3.5 μm particles. Peptides were eluted at a flow rate of 230 μL/min with a gradient of 5% buffer B (20 mM ammonium formate in 80% acetonitrile, pH 10) for 5 min, 5% to 15% buffer B for 15 min, 15% to 25% buffer B for 10 min, 25% to 55% buffer B for 10 min, and 55% to 95% buffer B for 5 min. The system was then maintained in 95% buffer B for 5 min before equilibrating with 5% buffer B for 10 min prior to the next injection. Elution was monitored by measuring the absorbance at 214 nm, and fractions were collected every 2 min. Fractions containing eluted peptides were pooled into 15 fractions based on peptide density^31^ and vacuum-dried for nano-ESI-LC-MS/MS analysis.

### LC-MS/MS analysis

LC-MS experiments were performed on a nano-flow HPLC system (Easy-nLC II, Thermo Fisher Scientific, Waltham, MA, USA) connected to a LTQ-Orbitrap Velos Pro (Linear quadrupole ion trap-Orbitrap mass analyser) mass spectrometer (Thermo Fisher Scientific), equipped with a Nanospray Flex Ion Source (Thermo Fisher Scientific). Peptide mixtures (5 μL) were injected at a flow rate of 5 μL/min onto a pre-column (Easy-column C18-A1, 100 μm I.D. × 20 mm, 5 μm, Thermo Fisher Scientific). Chromatographic separation was performed on a reversed phase C18 column (Easy-column C18-A2, 75 μm I.D. × 100 mm, 3 μm, Thermo Fisher Scientific) at a flow rate of 300 nL/min with a 60 min gradient of 2% to 40% acetonitrile in 0.1% formic acid. The electrospray voltage was maintained at 2.2 kV, and the capillary temperature was set at 250 °C. The LTQ-Orbitrap was operated in data-dependent mode to simultaneously measure full scan MS spectra (m/z 350–2000) in the Orbitrap with a mass resolution of 60,000 at m/z 400. After full-scan survey, the 15 most abundant ions detected in the full-MS scan were measured in the LTQ-Orbitrap using collision-induced dissociation (CID).

### Protein identification and quantitation

Data analysis was performed with MaxQuant software^32^ version 1.4.1.2 (http://www.maxquant.org/). For protein identification, MS/MS data were submitted to the UniProt human protein database (release 3.43, 72,340 sequences) using the Andromeda search engine^33^ with the following settings: trypsin cleavage; fixed modification of carbamidomethylation of cysteine; variable modifications of methionine oxidation; a maximum of two missed cleavages; false discovery rate calculated by searching the decoy database. Other parameters were set as default. The results were imported into Microsoft excel for further analysis.

Label-free quantitation (LFQ) was also performed in MaxQuant. The Minimum ratio count for LFQ was set to 2, and the match-between-runs option was enabled. Other parameters were set as default. Up- and down-regulated proteins were defined based on a significantly altered protein ratio (p-values < 0.01). Significance p-values were calculated using Perseus software (version 1.4.1.3; http://www.perseus-framework.org). A 2-fold change in expression and a p-value of 0.01 were used as a combined threshold to define biologically regulated proteins.

### Network analysis

Protein-protein interaction networks were constructed from data with or without B12 treatment using the BisoGenet^34^ plugin in the Cytoscape environment. Differentially expressed proteins were used as initial query terms. Experimentally supported hyperlinks from DIP, BIOGRID, HPRD, BIND, MINT and INTACT were then automatically connected using the SysBiomics platform and visualized in Cytoscape. The MCODE (version 1.4.0-beta2) plugin was used to find clusters (highly interconnected regions) in the resulting networks^35^. The search parameters were set as follows: network scoring degree cut-off = 2; cluster finding node score cut-off = 0.2; k-core = 2; maximum depth = 100; singly connected nodes were removed from clusters. The BiNGO plugin^36^ in the Cytoscape environment was used to retrieve the Gene Ontology Consortium (GOC, http://geneontology.org/).

### Western blotting

Treated cells (1 × 10^7^ cells/10 ml in 100 mm dishes) were collected and washed with PBS. After centrifugation (1000rpm, 5 min, room temperature), cells were lysed in RIPA buffer (Applygen, Beijing, China) and protein concentration was determined by BCA assay (Pierce, Thermo Fisher Scientific, MA, USA). After the addition of sample loading buffer, protein samples were separated using 12% SDS-PAGE and subsequently transferred to a nitrocellulose membrane (Amersham Pharmacia Biotech, UK). The membrane was incubated in fresh blocking buffer (0.1% Tween 20 in Tris-buffered saline, pH 7.4, containing 5% non-fat dried milk) at room temperature for 30 min and then probed with monoclonal mouse anti-PTBP1 antibody (Abcam, Cambridge, UK) and mouse anti-glyceraldehyde-3-phosphate dehydrogenase (GAPDH) antibody (Shanghai Kangchen Biotechnology Co. Ltd, China) in blocking buffer at 4 °C overnight. The membrane was washed three times for 5 min each using PBST (PBS containing 0.1% Tween-20), incubated with the appropriate horseradish peroxidase (HRP)-conjugated secondary antibody at room temperature for 1 h, and washed three more times in PBST buffer. The membrane was finally incubated with ECL substrate solution (Shanghai Pufei Biotechnology Co., Ltd, China) for 5 min according to the manufacturer’s instructions, and visualized with autoradiographic film.

### Down-regulation of PTBP1 by siRNA

PTBP1 small interfering RNA (PTBP1 siRNA; Abnova) and negative control siRNA were transiently transfected into SH-SY5Y cells using siRNA transfection medium (Opti-MEM) and siRNA transfection reagent (lipofectin 2000) according to the manufacturer’s instructions. Primer sequences were as follows: Sense = CCUCUAGAGUGAU (ccacatcc); Antisense = (atgtgg) AUCACUCUAGAGGGG. In brief, 2 × 10^6^ cells were seeded in 10 cm plates in fresh culture medium and cultured for 24 h. Transfection medium containing siRNA and siRNA transfection reagent was mixed with non-FBS culture medium and added to cells. Culture medium was replaced after 12 h, and cells were incubated for further 72 h. Cells were collected and lysed, and PTBP1 expression was then measured. To assess cell viability, cells were incubated for a further 36 h after transfection and treatment with B12 and H_2_O_2_.

### Statistical analysis

Results are expressed as mean ± S.E.M. Statistical evaluation was performed using the Student’s *t*-test (for comparing two value sets). P < 0.05 was considered statistically significant (*P < 0.05: **P < 0.01).

### Data availability

Mass spectrometry proteomics data have been deposited at the ProteomeXchange Consortium (http://proteomecentral.proteomexchange.org) via the MassiVE partner repository with the dataset identifier PXD003391.

## Additional Information

**How to cite this article**: Zhong, L. *et al*. Quantitative proteomics study of the neuroprotective effects of B12 on hydrogen peroxide-induced apoptosis in SH-SY5Y cells. *Sci. Rep.* **6**, 22635; doi: 10.1038/srep22635 (2016).

## Supplementary Material

Supplementary Information

Supplementary Table S1

Supplementary Table S2

## Figures and Tables

**Table 1:** Identification of differentially expressed proteins in cells treated with H_2_O_2_ + B12 (B12) compared with H_2_O_2_ alone (Ctrl) using LC-MS/MS.

Protein ID	Protein name	Gene name	Ratio (B12/Ctrl)	−log_10_ t-test p-value
P04733	Metallothionein-1F; Metallothionein	*MT1F*	9.9526	2.3245
P55854	Small ubiquitin-related modifier 3	*SUMO3*	7.8273	2.6933
Q9ULX6	A-kinase anchor protein 8-like	*AKAP8L*	4.511	2.0557
D6REN3	Proteasome assembly chaperone 4	*PSMG4*	3.2784	3.0269
P00374	Dihydrofolate reductase	*DHFR*	2.9365	2.2306
J3QKT4	Pyrroline-5-carboxylate reductase; Pyrroline-5-carboxylate reductase 1, mitochondrial	*PYCR1*	2.832	2.1511
Q96Q83	Alpha-ketoglutarate-dependent dioxygenase alkB homolog 3	*ALKBH3*	2.3276	2.9638
Q96G46-3	tRNA-dihydrouridine (47) synthase [NAD(P)( + )]-like	*DUS3L*	2.1294	2.1323
K7ELW5	Polypyrimidine tract binding protein 1	*PTBP1*	2.1231	3.3389
C9JLV4	Apoptotic protease-activating factor 1	*APAF1*	2.06	2.0984
Q58FF6	Putative heat shock protein HSP 90-beta 4	*HSP90AB4P*	2.0514	2.1456
Q9NV56	MRG/MORF4L-binding protein	*MRGBP*	1.9973	2.0082
P14314	Glucosidase 2 subunit beta	*PRKCSH*	0.4952	2.3677
Q07666	KH domain-containing, RNA-binding, signal transduction-associated protein 1	*KHDRBS1*	0.4359	2.3355
F8WAS3	NADH dehydrogenase [ubiquinone] 1 alpha subcomplex subunit 5	*NDUFA5*	0.4257	2.4447
Q9NQW6	Actin-binding protein anillin	*ANLN*	0.4158	2.0437
Q5TA45	Integrator complex subunit 11	*CPSF3L*	0.2806	2.4838
F6XY72	Nucleoside diphosphate kinase	*NME2*	0.2688	2.1652
P05997	Collagen alpha-2 (V) chain	*COL5A2*	0.2678	2.9118
Q5QJ74	Tubulin-specific chaperone cofactor E-like protein	*TBCEL*	0.2513	3.1746
P15924	Desmoplakin	*DSP*	0.1897	2.8376
Q9P2 × 0	Dolichol phosphate mannosyltransferase subunit 3	*DPM3*	0.038	2.263
