# Supplementary material for: Quantitative proteomics study of the neuroprotective effects of B12 on hydrogen peroxide-induced apoptosis in SH-SY5Y cells
Source: Sci Rep. 2016 Mar 8;6:22635. doi: 10.1038/srep22635 (PMC4782069; doi:10.1038/srep22635)
Supplement: Supplementary Information [file srep22635-s1.pdf]

Supplementary Information For:

Quantitative proteomics study of the neuroprotective effects of B12 on hydrogen peroxide-induced apoptosis in SH-SY5Y cells

Lijun Zhong [a,#], Juntuo Zhou [b,#] , Xi Chen [a], Yaxin Lou [a], Dan Liu [a], Xiajuan Zou [a], Bin Yang [a], Yuxin Yin[c,d,*], Yan Pan[e,*]

[a] *Medical and Health Analytical Center, Peking University Health Science Center, Beijing 100191, China*

[b] *Department of Pathology, School of Basic Medical Sciences, Peking University Health Science Center, Beijing 100191,China*

[c] *Institute of Systems Biomedicine, Department of Pathology, School of Basic Medical Sciences, Peking University Health Science Center, Beijing, 100191, China.*

[d] Beijing Key Laboratory of Tumor Systems Biology, Beijing, 100191, China

[e] *Department of Pharmacology, School of Basic Medical Sciences, Peking University Health Science Center, Beijing 100191, China*

*#Co-first authors，*Co-corresponding authors*

Corresponding author:

Yan Pan

Address: *Department of Pharmacology, School of Basic Medical Sciences, Peking University Health Science Center, Beijing 100191, China*

Tel.: +86-10-82801890

E-mail address: **pannay26@bjmu.edu.cn**

Supplementary figures and figure legends

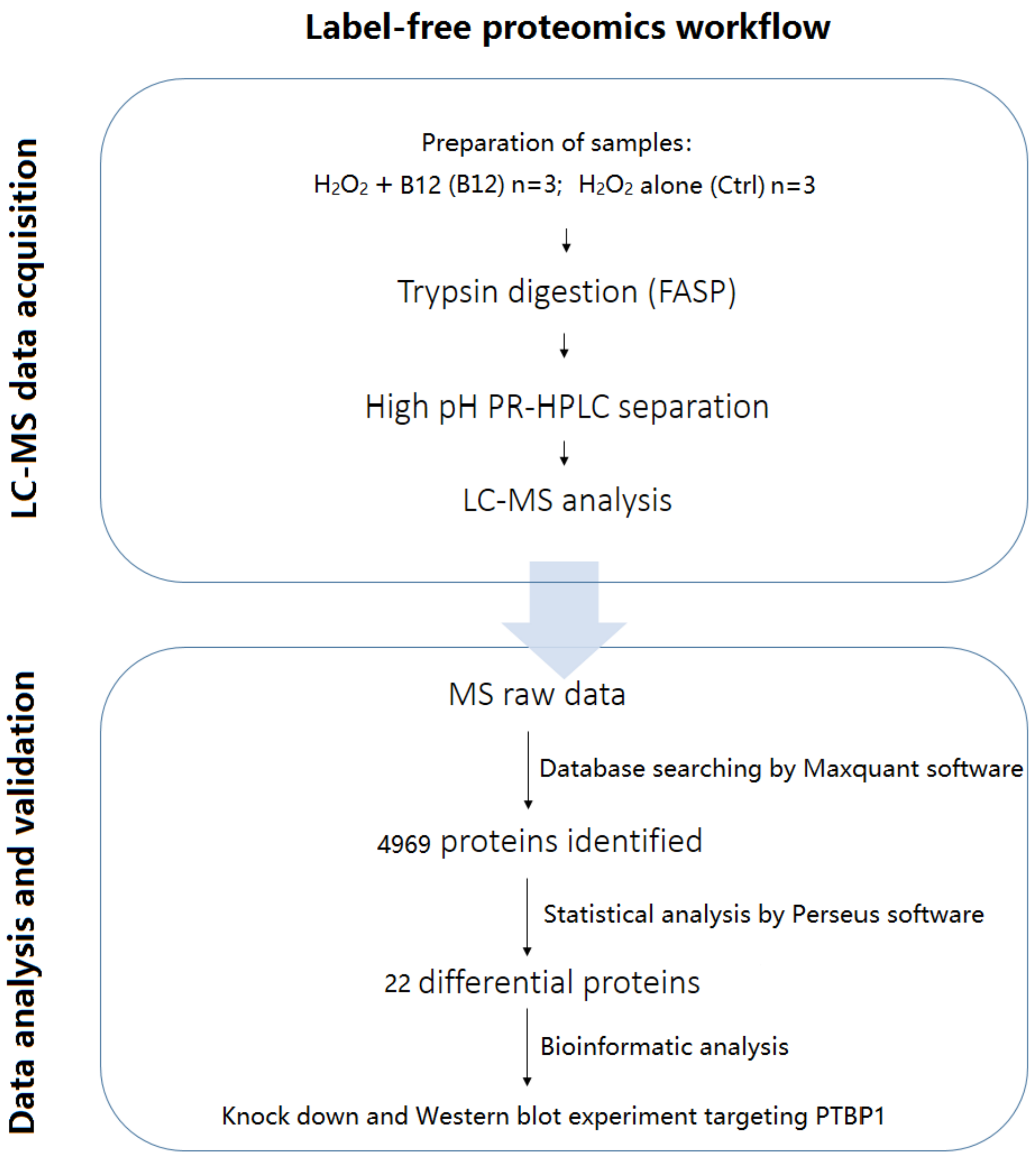

Figure S1 |. The workflow flowchart of the proteomic study.

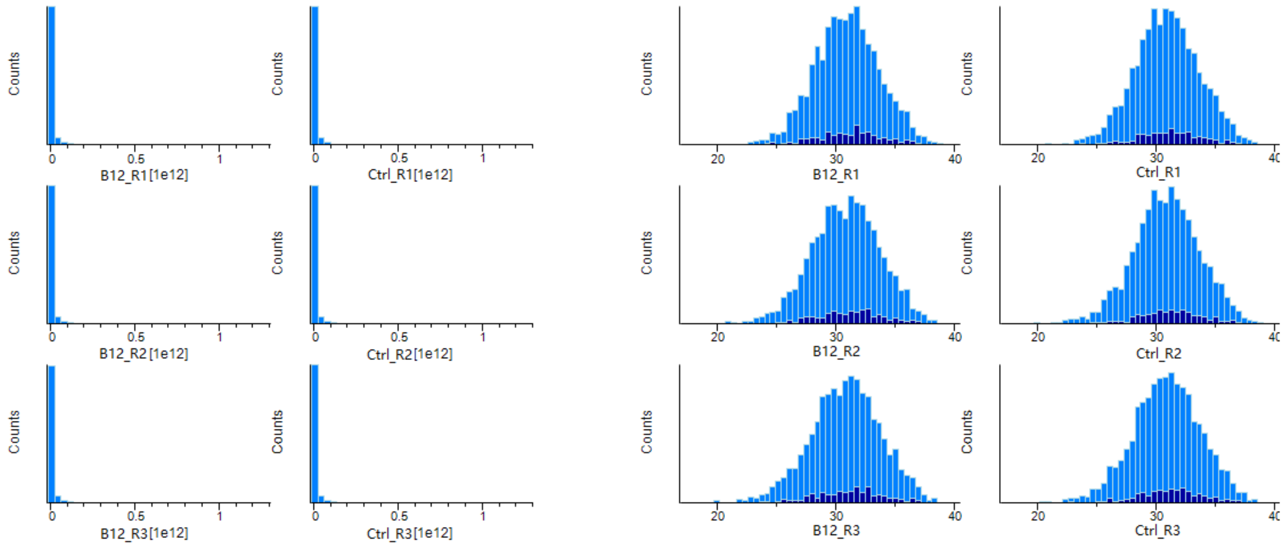

Figure S2 |.Histograms of the LFQ intensity of proteins before (left) and after (right) a logarithm transformation (base 2). Blue represents total proteins identified in SH-SY5Y cells and dark blue represents proteins that had a t-test p-value less than 0.05 between the two groups (B12 and Ctrl group).

List names	number of elements	number of unique elements
Bioreplicate_1	4215	4215
Bioreplicate_2	4331	4331
Bioreplicate_3	4546	4546
**Overall number of unique elements**		**4958**

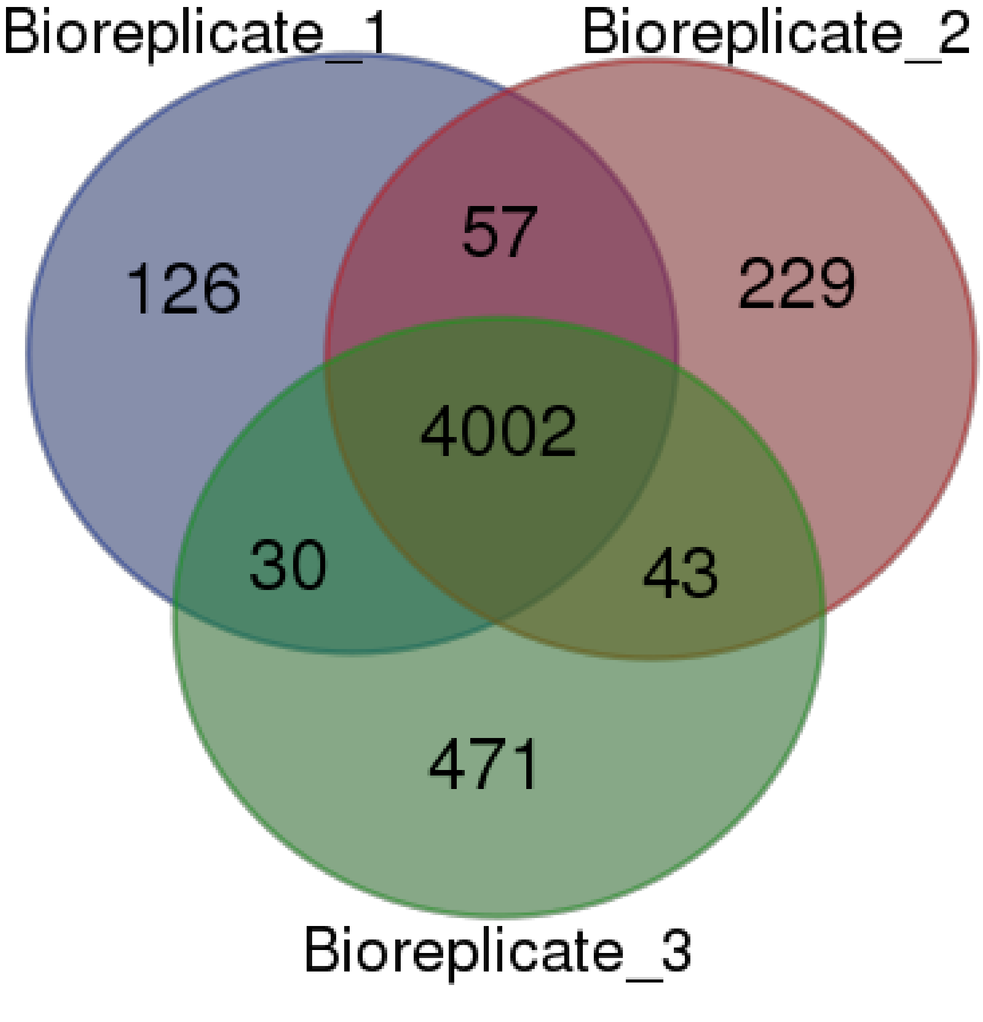

Figure S3 |.Venn diagram of identified proteins in the three biological replicates.

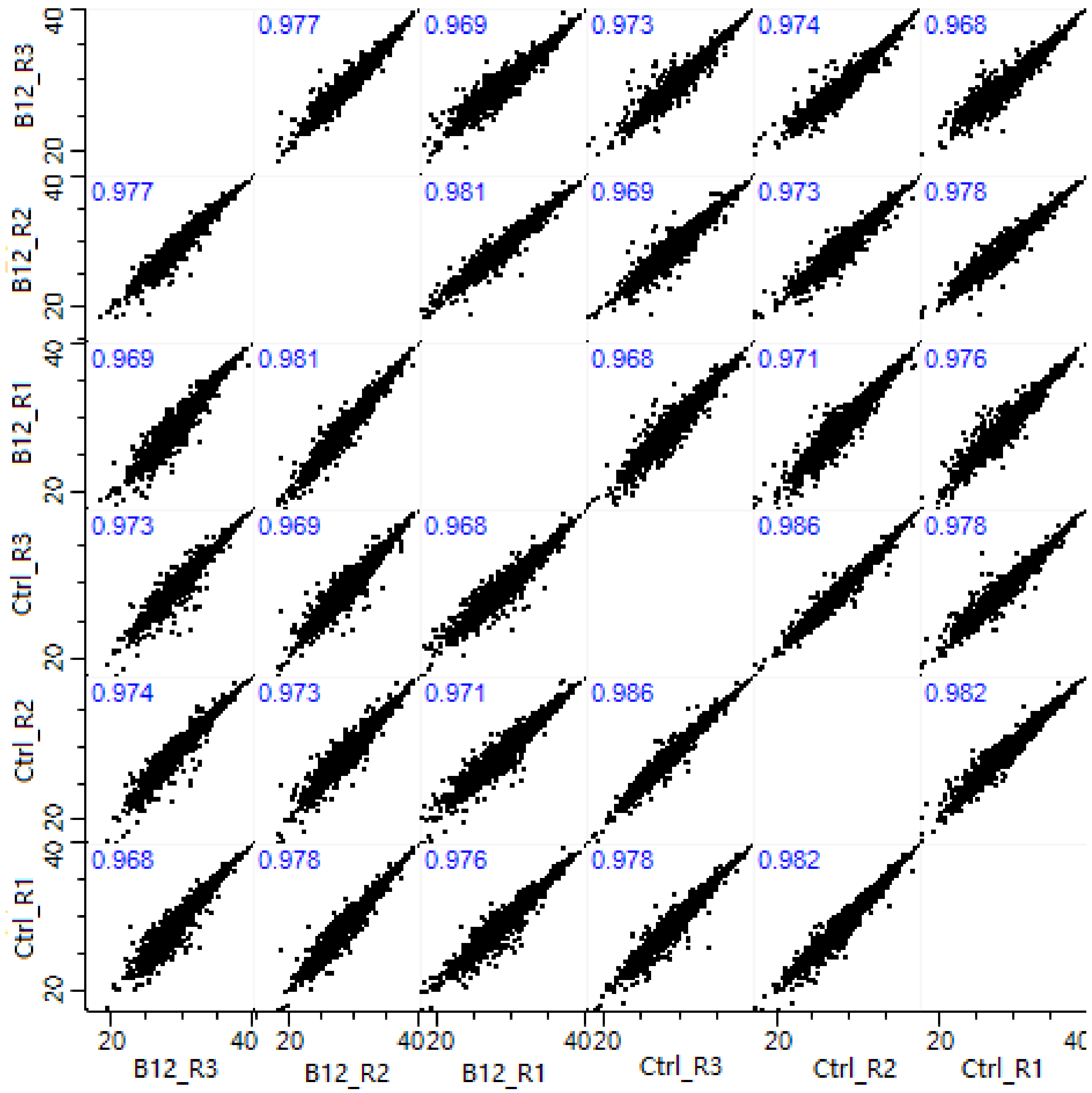

Figure S4 |.Scatter plots of log2 LFQ intensities of identified proteins of all samples. The number in plots was the Pearson correlation coefficient.
